# Pathogenicity, Ovicidal Action, and Median Lethal Concentrations (LC_**50**_) of Entomopathogenic Fungi against Exotic Spiralling Whitefly, *Aleurodicus dispersus* Russell

**DOI:** 10.1155/2013/393787

**Published:** 2013-12-23

**Authors:** Boopathi Thangavel, Karuppuchamy Palaniappan, Kalyanasundaram Manickavasagam Pillai, Mohankumar Subbarayalu, Ravi Madhaiyan

**Affiliations:** ^1^Division of Agricultural Entomology, ICAR Research Complex for NEH Region, Mizoram Centre, Kolasib, Mizoram 796081, India; ^2^Department of Agricultural Entomology, Centre for Plant Protection Studies, Tamil Nadu Agricultural University, Coimbatore, Tamil Nadu 641 003, India; ^3^Department of Plant Biotechnology, Centre for Plant Molecular Biology and Biotechnology, Tamil Nadu Agricultural University, Coimbatore, Tamil Nadu 641 003, India; ^4^Krishi Vigyan Kendra, Tamil Nadu Agricultural University, Sirugamani, Tiruchirappalli, Tamil Nadu 641115, India

## Abstract

Biological control using entomopathogenic fungi could be a promising alternative to chemical control. Entomopathogenic fungi, *Beauveria bassiana* (Balsamo) Vuillemin, *Metarhizium anisopliae* (Metschnikoff) Sorokin, *Lecanicillium lecanii* (Zimmerm.) Zare and Gams, and *Paecilomyces fumosoroseus* (Wize) Brown and Smith, were tested for their pathogenicity, ovicidal effect, and median lethal concentrations (LC_50_) against exotic spiralling whitefly, *Aleurodicus dispersus* Russell. The applications were made at the rate of 2 × 10^9^ conidia mL^−1^ for evaluating the pathogenicity and ovicidal effect of entomopathogenic fungi against *A. dispersus*. The results of pathogenicity test showed that *P. fumosoroseus* (P1 strain) was highly pathogenic to *A. dispersus* recording 100% mortality at 15 days after treatment (DAT). *M. anisopliae* (M2 strain) had more ovicidal effect causing 37.3% egg mortality at 8 DAT. However, *L. lecanii* (L1 strain) caused minimum egg hatchability (23.2%) at 10 DAT as compared to control (92.6%). The lowest LC_50_ produced by *P. fumosoroseus* (P1 strain) as 8.189 × 10^7^ conidia mL^−1^ indicated higher virulence against *A. dispersus*. Hence, there is potential for use of entomopathogenic fungi in the field conditions as an alternate control method in combating the insect pests and other arthropod pests since they are considered natural mortality agents and are environmentally safe.

## 1. Introduction

The spiralling whitefly, *Aleurodicus dispersus* Russell (Homoptera, Aleyrodidae), the native of the Caribbean region of Central America [1], is a highly polyphagous pest, which has extensive host range covering 481 plants belonging to 295 genera from 90 families of vegetables, fruits, and ornamentals trees [2]. A loss of 80% in fruit yield recorded in guava infested by *A. dispersus* in Taiwan [3] and *A. dispersus* caused yield reduction up to 53% in cassava [4]. The nymphs are covered with heavy waxy flocculent materials and waxy threads offering a great defense against synthetic chemical insecticides and resulting in poor control of the pest [5].

One of the potential methods in *A. dispersus *management is the use of microbial biocontrol agents (MBCAs) as the natural enemies of the pest population devastate pests with no hazard effects on human health and environment. As the microbial biocontrol agents have complex mode of action, it is very difficult for a pest to develop resistance against MBCAs. The present MBCAs are viruses, bacteria, nematodes, and fungi and they are used throughout the world with great advantage and success. But fungal biocontrol agents are the most important among all the MBCAs due to easy delivery, improving formulation, vast number of pathogenic strains known, easy engineering techniques, and overexpression of endogenous proteins or exogenous toxins [6–8]. Similarly, the entomopathogenic fungi are important among all the biological control agents due to their broad host range, route of pathogenicity and their ability to control sap sucking pests such as mosquitoes and aphids [9–11] as well as pests with chewing mouthparts [12, 13]. With this background, the research on entomopathogenic fungi was conducted to assess the pathogenicity, ovicidal effect, and median lethal concentrations (LC_50_) of entomopathogenic fungi against *A. dispersus* under laboratory conditions.

## 2. Materials and Methods

Studies were conducted at Biocontrol Laboratory, Department of Agricultural Entomology, Centre for Plant Protection Studies, Tamil Nadu Agricultural University, Coimbatore, Tamil Nadu, India. Bioassays were carried out to evaluate the pathogenicity, ovicidal effect, and LC_50_ of entomopathogenic fungi. The insect used for the study was *A. dispersus* from the culture maintained in screen house.

### 2.1. Fungal Isolates and Culture Maintenance

The entomopathogenic fungi, *B. bassiana *(B1, B2 strains), *M. Anisopliae* (M1, M2, M3 strains), *L. lecanii* (L1 strain), and *P. fumosoroseus* (P1 strain) were obtained from the National Bureau of Agriculturally Important Insects, Bangalore, Karnataka, India, the Department of Plant Pathology, Tamil Nadu Agricultural University (TNAU), Coimbatore, Tamil Nadu, India, and Sun Agro Biotech Research Centre, Porur, Chennai, Tamil Nadu, India, culture collections. Isolates were maintained in culture on potato dextrose agar (PDA) slants in universal bottles (30 mL) and stored at 4°C. Continuous cultures were maintained on slants with subcultures grown for 14 days at 25°C following which lids were tightly sealed and cultures stored at 4°C.

### 2.2. Pathogenicity of Entomopathogenic Fungi against *A. dispersus*

Strains of different entomopathogenic fungi were assayed against *A. dispersus* nymphs by direct spray method in completely randomized design (CRD). Entomopathogenic fungi were sprayed with the help of automizer over the nymphs of *A. dispersus *with three replications. All the treated Petri dishes were maintained at 25 ± 1°C in an incubator. The nymphs were individually examined under a stereo zoom binocular microscope (Carl Zeiss Stemi 2000) at 40x magnification for verification of fungal infection. The mortality data were recorded by counting the dead cadavers and nymphs with fungal spores. Observations on the mortality of *A. dispersus *by entomopathogenic fungi were made at 3, 5, 7 10, 13, and 15 days after treatment (DAT). The mortality data were corrected using Abbott's formula [14]. The experiments were repeated for three times to confirm the pathogenicity of entomopathogenic fungi against *A. dispersus*.

### 2.3. Ovicidal Effect of Entomopathogenic Fungi against *A. dispersus*

Entomopathogenic fungi were assayed using direct spray method to evaluate ovicidal effect on *A. dispersus* eggs. Uniform age of *A. dispersus *eggs were taken from eggplant (*Solanum melongena* L.) leaf placed on 1.5% agar in a Petri dish. Entomopathogenic fungi were sprayed with help of automizer over the eggs of *A. dispersus *with three replications in CRD. All the treated Petri dishes were maintained at 25 ± 1°C in an incubator and hatchability was recorded until no change for three consecutive days. Later, the eggs were individually examined under a stereo zoom binocular microscope (Carl Zeiss Stemi 2000) at 40x magnification for verification of fungal infection. Finally, all unhatched eggs were transferred to moist chambers for three days to observe fungal outgrowth if any, as an evidence of egg mortality due to fungal infection. Observations were made at 4, 6, 8, and 10 DAT. The experiments were repeated for three times to confirm the ovicidal action of entomopathogenic fungi against *A. dispersus* eggs.

### 2.4. Median Lethal Concentrations (LC_**50**_) of Entomopathogenic Fungi against *A. dispersus* Nymphs

Studies were conducted to find the median lethal concentrations (LC_50_) of four entomopathogenic fungi, namely, *M. anisopliae *(M1 strain), *B. bassiana *(B1 strain), *L. lecanii* (L1 strain), and *P. fumosoroseus* (P1 strain), against *A. dispersus* nymphs. Five doses (from 2 × 10^5^ to 2 × 10^9^ conidia mL^−1^) were fixed for which dilutions were prepared with double distilled water. *A. dispersus *nymphs were treated starting from lower to higher concentrations, whenever different test doses the of same entomopathogenic fungi were used. Uniform age of *A. dispersus *nymphs was taken from eggplant leaf placed on 1.5% agar in a Petri dish. Five concentrations of each respective entomopathogenic fungi were sprayed with the help of automizer over the *A. dispersus *nymphs with three replications in CRD. All the treated Petri dishes were maintained at 25 ± 1°C in an incubator. The nymphs were individually examined under a stereo zoom binocular microscope (Carl Zeiss Stemi 2000) at 40x magnification for verification of fungal infection. The mortality data were recorded by counting the dead cadavers and nymphs with fungal spores. Observations were made periodically at 12 h interval up to 14 days and mortality data were recorded and corrected using Abbott's formula [14]. The median lethal concentrations (LC_50_) and LC_95_ values were estimated for *A. dispersus* [15].

### 2.5. Data Analysis

Statistical analysis was done in completely randomized design. The percentage of mortality in both eggs and nymphs was collected and corrected with that in control by using Abbott's formula [14] as follows:

(1) $$\begin{array}{c} P=\left[\frac{C-T}{C}\right]\times 100, \end{array}$$

where *P* = estimated percentage of insects killed by fungus alone, *C* = percentage of control insects living, and *T* = percentage of treated insects that are living after the experimentation period.

## 3. Results and Discussion

### 3.1. Pathogenicity of Entomopathogenic Fungi

**Figure 1:**
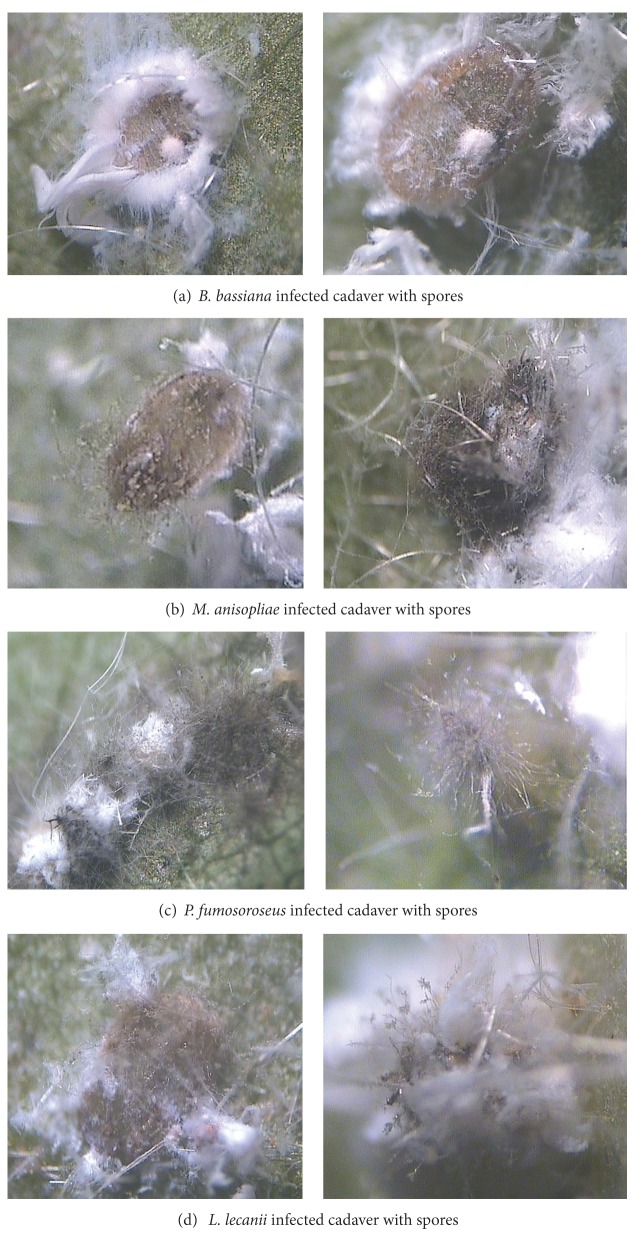
Infected cadavers of *A. dispersus *by entomopathogenic fungi.

**Figure 2:**
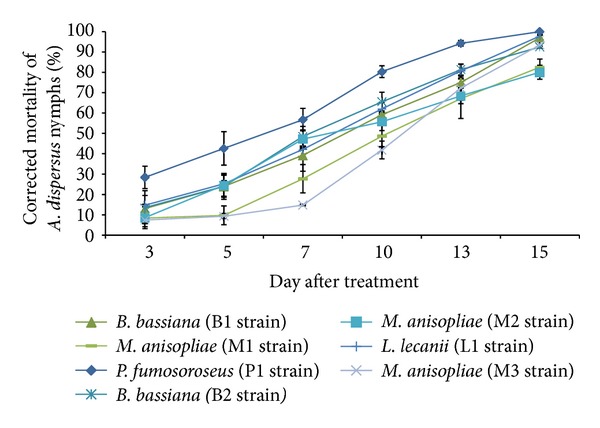
Pathogenicity of entomopathogenic fungi against *A. dispersus *nymphs.

All entomopathogenic fungi caused high rates of pathogenicity among *A. dispersus* population. *A. dispersus* population infected by *B. bassiana *was distinctly red to red brown. Hyphal growth and sporulation of *P. fumosoroseus *were visibly greater and more rapid than those of the other entomopathogenic fungi (Figure 1). The results of pathogenicity test against *A. dispersus* revealed that *P. fumosoroseus *(P1 strain) caused significantly maximum mortality (80.4%) at 10 DAT as compared to other entomopathogenic fungi isolates (Figure 2). *P. fumosoroseus *(P1 strain) produced 100% mortality to *A. dispersus* nymphs. The next best entomopathogenic fungi were *L. lecanii* (L1 strain) (97.8%) and *B. bassiana *(B1 strain) (97.0%) at 15 DAT. Earlier, Avery et al. [16] reported that *P. fumosoroseus* produced 99.5% mortality to greenhouse whitefly, *Trialeurodes vaporariorum* (Westwood), and this is in conformity with the present findings. *Paecilomyces* isolates produced over 70% mortality to *T. vaporariorum* as reported by Gökçe and Er [17]. Wraight et al. [18] recorded the pathogenicities of three species of entomopathogenic fungi (*P. fumosoroseus*, *P. farinosus,* and *Beauveria bassiana*) against silver leaf whitefly, *Bemisia argentifolii *Bellows and Perring. Eyal et al. [19] reported 52–98% mortality of *Bemisia tabaci* (Gennadius) by *B. bassiana *with concentrations of 1–4 × 10^6^ conidia mL^−1^. Nagasi et al. [20] observed that *B. bassiana* was most pathogenic to first instar and adults of *B. argentifolii*. However, Wraight and Knaf [21] and Wraight et al. [22] reported higher dose of 5 × 10^13^ conidia (2.5 conidia mL^−1^) and achieved 90% control of *B. tabaci* nymphs on 7 DAT.

### 3.2. Ovicidal Effect of Entomopathogenic Fungi

**Figure 3:**
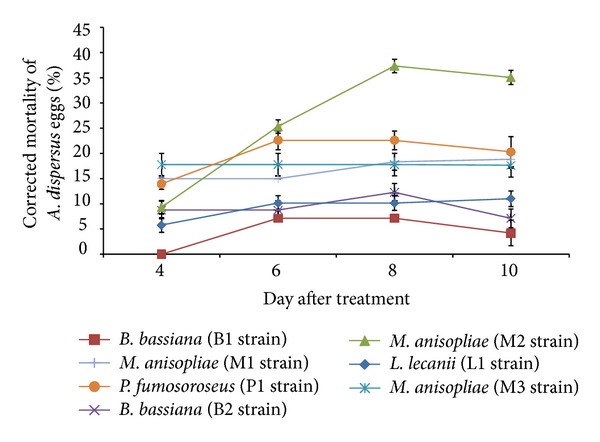
Ovicidal action of entomopathogenic fungi against *A. dispersus *eggs.

**Figure 4:**
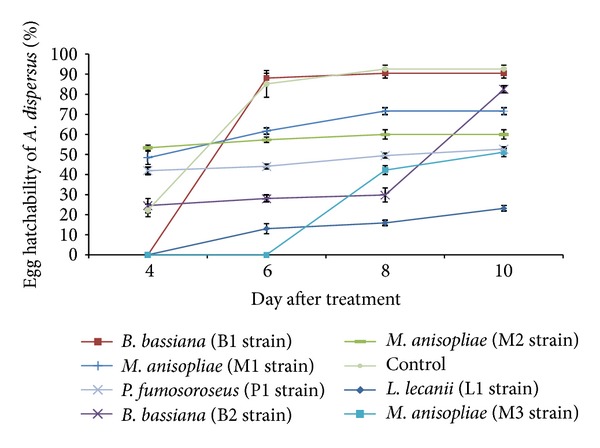
Effect of entomopathogenic fungi on egg hatchability of *A. disperses*.

*M. anisopliae *(M2 Strain) caused 37.3% egg mortality and the next higher egg mortality was with *P. fumosoroseus *(P1 strain) (22.6%) at 8 DAT (Figure 3). Very low ovicidal effect was observed in *B. bassiana* (B1 strain) (4.2%). The hatchability was suppressed by all the entomopathogenic fungi to some extent (Figure 4). *L. lecanii* (L1 strain) produced lesser egg hatchability (23.2%) at 10 DAT as compared to other fungi. Earlier, Pirali-Kheirabadi et al. [23] reported remarkable effects of *M. anisopliae*, *B. bassiana,* and *Lecanicillium psalliotae* (Treschew) Zare and W. Gams on egg hatchability of *Rhipicephalus* (*Boophilus*) *annulatus *(Say). However, Malarvannan et al. [24] reported that *B. bassiana* at 2.4 × 10^7^ conidia mL^−1^ did not affect the hatchability of *Spodoptera litura* Fabricius.

### 3.3. Median Lethal Concentrations (LC_**50**_) of Entomopathogenic Fungi against *A. dispersus*

**Figure 5:**
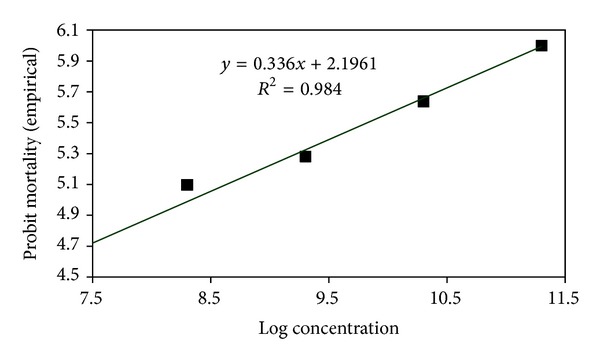
Log concentration probit mortality response of *A. dispersus *to *M. anisopliae *(M1 strain).

**Figure 6:**
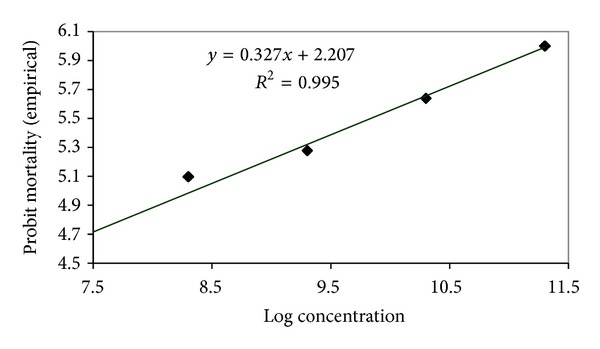
Log concentration probit mortality response of *A. dispersus *to *B. bassiana *(B1 strain).

**Figure 7:**
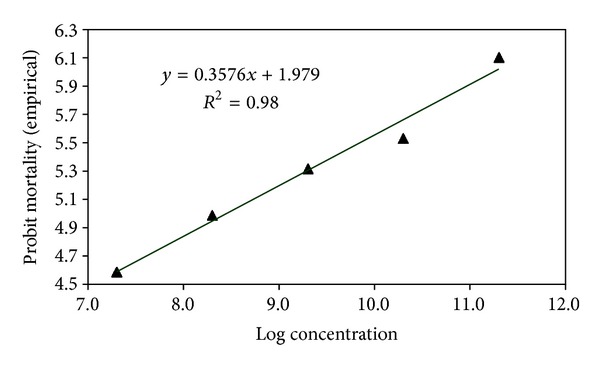
Log concentration probit mortality response of *A. dispersus *to *L. lecanii *(L1 strain).

**Figure 8:**
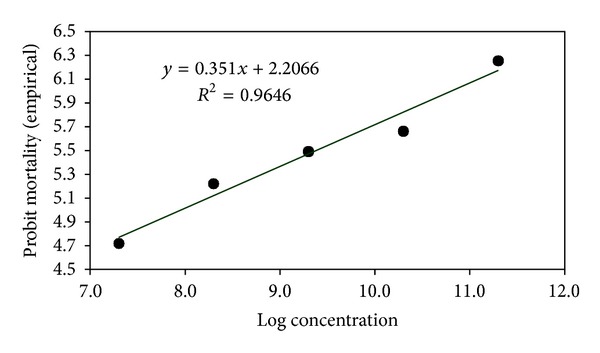
Log concentration probit mortality response of *A. dispersus *to *P. fumosoroseus *(P1 strain).

The LC_50_ of *L. lecanii *(L1 strain), *P. fumosoroseus *(P1 strain), *M. anisopliae* (M1 strain) and *B. bassiana* (B1 strain) assessed for *A. dispersus *population were 3.085 × 10^8^, 8.189 × 10^7^, 2.197 × 10^8^, and 3.481 × 10^8^ conidia mL^−1^, respectively (Table 1). The LC_95_ of *L. lecanii *(L1 strain), *P. fumosoroseus *(P1 strain), *M. anisopliae* (M1 strain), and *B. bassiana* (B1 strain) assessed for *A. dispersus *population were 2.513 × 10^13^, 5.053 × 10^12^, 1.506 × 10^13^, and 3.442 × 10^13^ conidia mL^−1^, respectively. Log concentration probit mortality response of *A. dispersus *to entomopathogenic fungi is depicted in Figures 5, 6, 7, and 8. In the present study, the lowest LC_50_ and LC_95_ were recorded by *P. fumosoroseus* as 8.189 × 10^7^ and 5.053 × 10^12^ conidia mL^−1^, respectively, indicating higher virulence against *A. dispersus*. Earlier, Saranya et al. [25] recorded the lowest LC_50_ value of 2.5 × 10^4^ spores mL^−1^ by *L. lecanii *and *Hirsutella thompsonii* Fisher isolates against cowpea aphid, *Aphis craccivora* (Koch). Low LC_50_ value of 1.2 × 10^4^ spores mL^−1^ for *L. lecanii *against Brevicoryne brassicae (L.) and 2.7 × 10^4^ spores mL^−1^ against *Aphis gossypii* Glover was reported by Derakshan et al. [26] and Karindah et al. [27], respectively. LC_50_ value obtained in the present study was lower than that reported by Smitha [28] for *Hirsutella *sp. (5.2 × 10^4^ spores mL^−1^) but higher than that reported by Liu et al. [29] for *B. bassiana *(1.2 × 10^4^ spores ml^−1^) and Chandler [30] for *M. anisopliae *(2.45 × 10^6^ spores mL^−1^). The difference in the LC_50_ values might be due to the difference in the virulence of fungal isolates and the host species.

Since they are considered natural mortality agents and are environmentally safe, there is potential for the use of entomopathogenic fungi in the field conditions as an alternate control method in combating the insect pests and other arthropod pests. Additional testing of entomopathogenic fungi with other stages of *A. dispersus *and field evaluation of entomopathogenic fungi must be conducted before ultimate conclusions are drawn.

## Figures and Tables

**Table 1:** LC_50_ and LC_95_ of entomopathogenic fungi against *A. dispersus*.

Entomopathogenic fungi	Regression equation	Calculated *χ* ^2^	LC_50_ (ppm)	Fiducial limits		LC_95_ (ppm)	Fiducial limits	
				Lower limit	Upper limit		Lower limit	Upper limit
*L. lecanii* (L1 strain)	*y* = 0.357*x* + 1.979	0.3176	3.085 × 10^8^	3.541 × 10^7^	2.688 × 10^9^	2.513 × 10^13^	5.562 × 10^10^	1.135 × 10^16^
*P. fumosoroseus* (P1 strain)	*y* = 0.351*x* + 2.206	0.3398	8.189 × 10^7^	4.926 × 10^6^	1.361 × 10^9^	5.053 × 10^12^	1.036 × 10^10^	2.465 × 10^15^
*M. anisopliae* (M1 strain)	*y* = 0.336*x* + 2.196	0.3493	2.197 × 10^8^	3.991 × 10^7^	1.209 × 10^9^	1.506 × 10^13^	6.926 × 10^10^	3.274 × 10^15^
*B. bassiana* (B1 strain)	*y* = 0.327*x* + 2.207	0.0448	3.481 × 10^8^	3.958 × 10^7^	3.061 × 10^9^	3.442 × 10^13^	9.624 × 10^10^	1.231 × 10^16^
